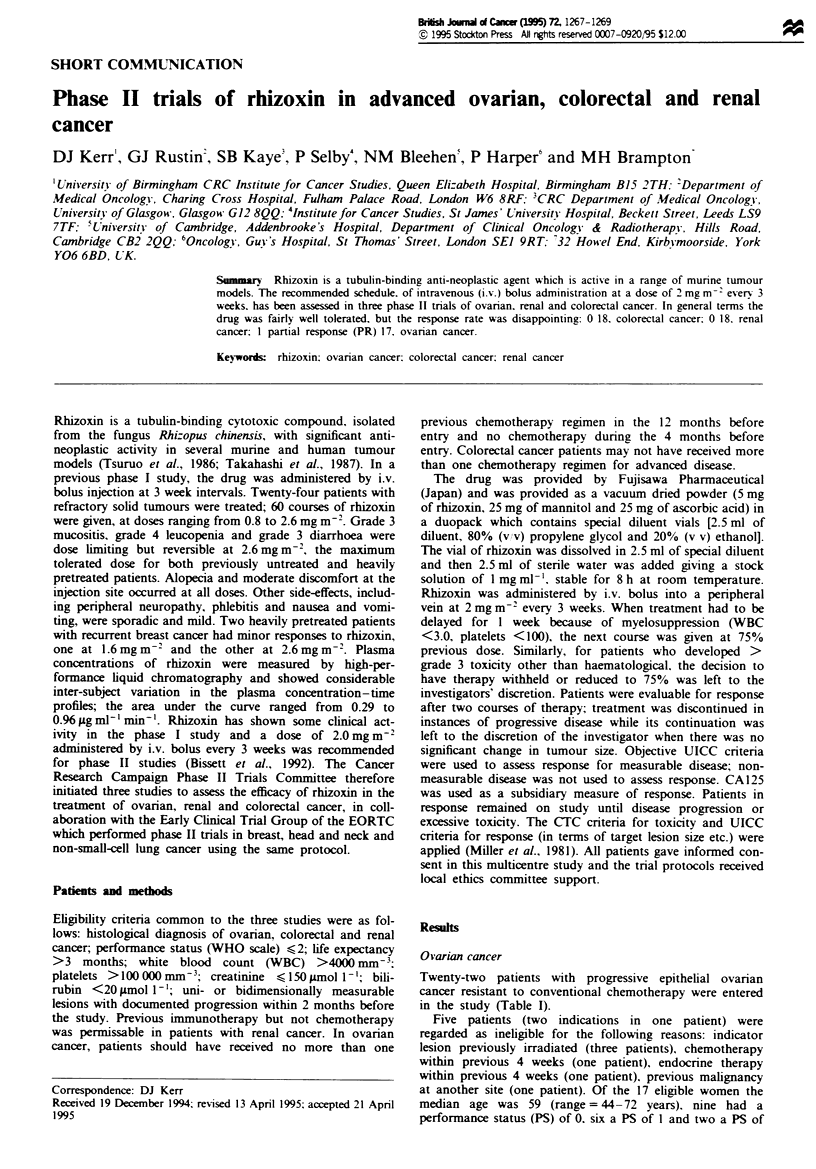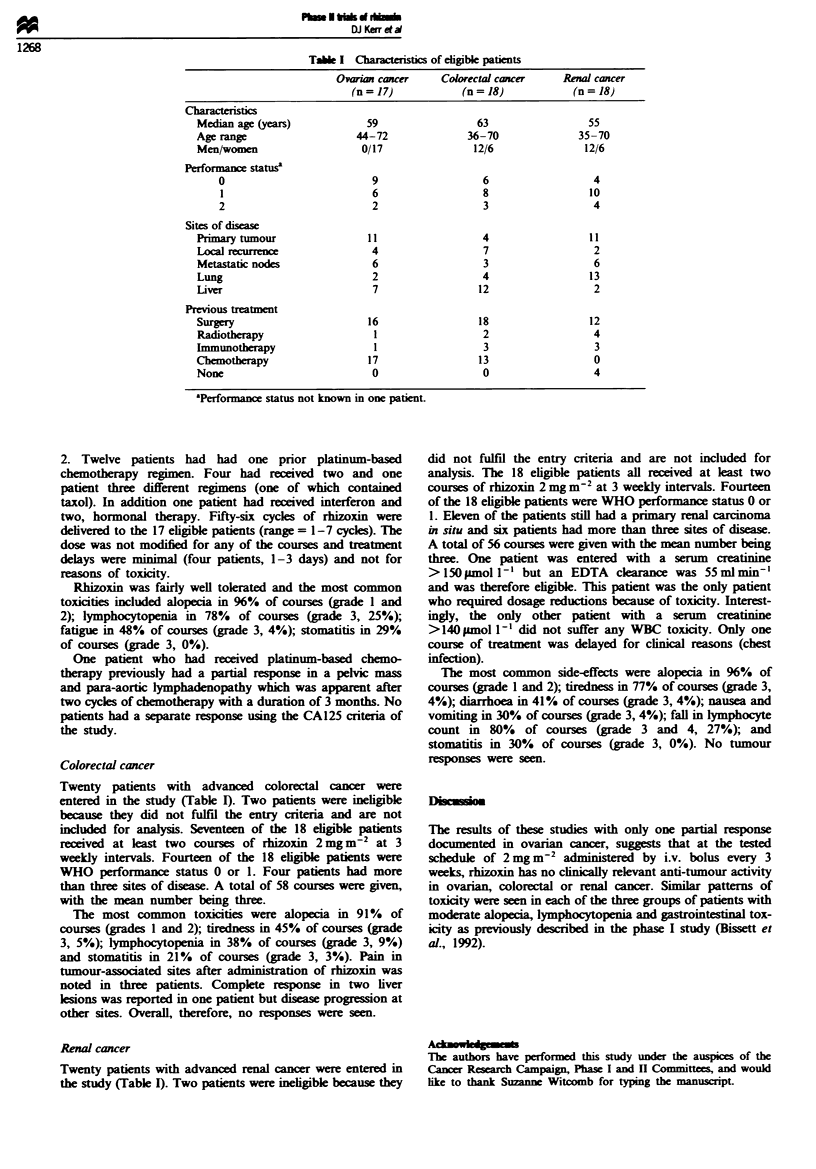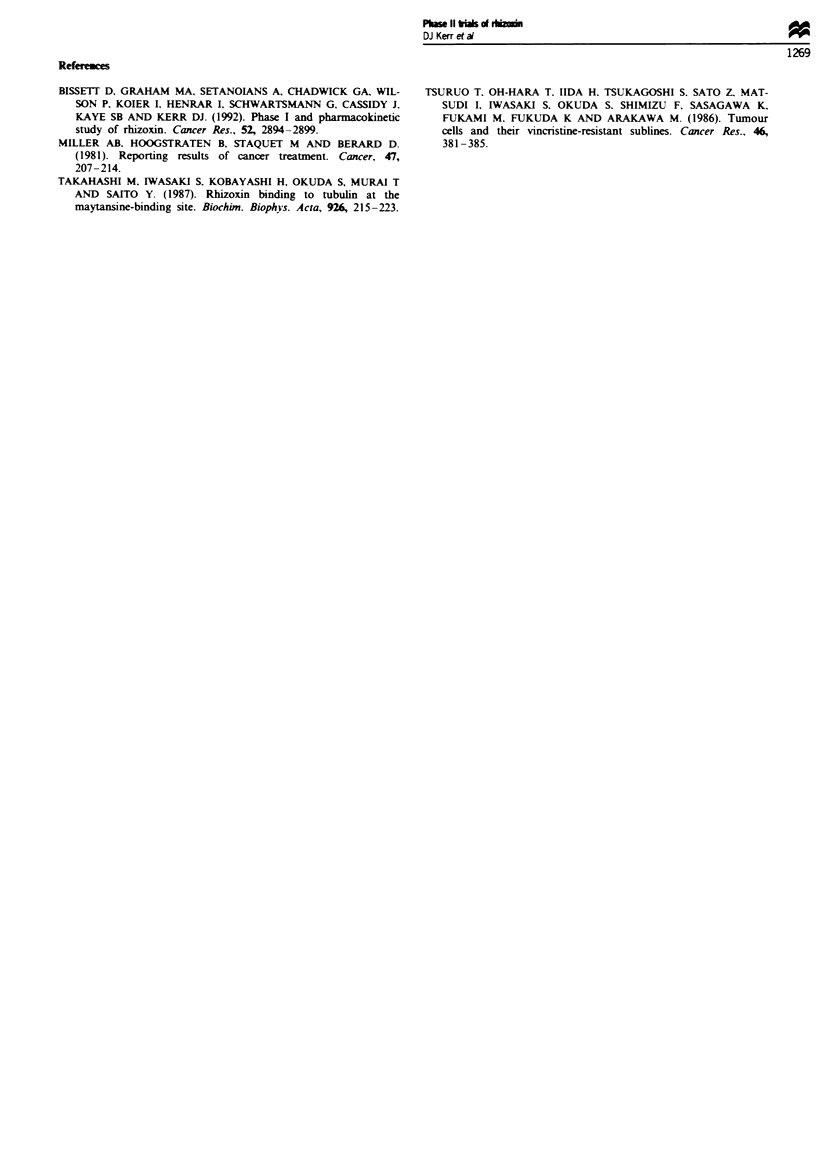# Phase II trials of rhizoxin in advanced ovarian, colorectal and renal cancer.

**DOI:** 10.1038/bjc.1995.498

**Published:** 1995-11

**Authors:** D. J. Kerr, G. J. Rustin, S. B. Kaye, P. Selby, N. M. Bleehen, P. Harper, M. H. Brampton

**Affiliations:** University of Birmingham CRC Institute for Cancer Studies, Queen Elizabeth Hospital, UK.

## Abstract

Rhizoxin is a tubulin-binding anti-neoplastic agent which is active in a range of murine tumour models. The recommended schedule, of intravenous (i.v.) bolus administration at a dose of 2 mg m-2 every 3 weeks, has been assessed in three phase II trials of ovarian, renal and colorectal cancer. In general terms the drug was fairly well tolerated, but the response rate was disappointing: 0/18, colorectal cancer; 0/18, renal cancer; 1 partial response (PR)/17, ovarian cancer.


British Journal of Cancer (1995) 72, 1267-1269

© 1995 Stockton Press All rights reserved 0007-0920/95 $12.00

SHORT COMMUNICATION

### Phase II trials of rhizoxin in advanced ovarian, colorectal and renal cancer

DJ Kerr[1], GJ Rustin[2], SB Kaye[3], P Selby[4], NM Bleehen[5], P Harper[6] and MH Brampton[7]

[1]*University of Birmingham CRC Institute for Cancer Studies, Queen Elizabeth Hospital, Birmingham B15 2TH;* [2]*Department of Medical Oncology, Charing Cross Hospital, Fulham Palace Road, London W6 8RF;* [3]*CRC Department of Medical Oncology, University of Glasgow, Glasgow G12 8QQ;* [4]*Institute for Cancer Studies, St James' University Hospital, Beckett Street, Leeds LS9 7TF;* [5]*University of Cambridge, Addenbrooke's Hospital, Department of Clinical Oncology & Radiotherapy, Hills Road, Cambridge CB2 2QQ;* [6]*Oncology, Guy's Hospital, St Thomas' Street, London SE1 9RT;* [7]*32 Howel End, Kirbymoorside, York YO6 6BD, UK.*

**Summary** Rhizoxin is a tubulin-binding anti-neoplastic agent which is active in a range of murine tumour models. The recommended schedule, of intravenous (i.v.) bolus administration at a dose of 2 mg m$^{-2}$ every 3 weeks, has been assessed in three phase II trials of ovarian, renal and colorectal cancer. In general terms the drug was fairly well tolerated, but the response rate was disappointing: 0 18, colorectal cancer; 0 18, renal cancer; 1 partial response (PR) 17, ovarian cancer.

**Keywords:** rhizoxin; ovarian cancer; colorectal cancer; renal cancer

Rhizoxin is a tubulin-binding cytotoxic compound, isolated from the fungus *Rhizopus chinensis*, with significant anti-neoplastic activity in several murine and human tumour models (Tsuruo *et al.*, 1986; Takahashi *et al.*, 1987). In a previous phase I study, the drug was administered by i.v. bolus injection at 3 week intervals. Twenty-four patients with refractory solid tumours were treated; 60 courses of rhizoxin were given, at doses ranging from 0.8 to 2.6 mg m$^{-2}$. Grade 3 mucositis, grade 4 leucopenia and grade 3 diarrhoea were dose limiting but reversible at 2.6 mg m$^{-2}$, the maximum tolerated dose for both previously untreated and heavily pretreated patients. Alopecia and moderate discomfort at the injection site occurred at all doses. Other side-effects, including peripheral neuropathy, phlebitis and nausea and vomiting, were sporadic and mild. Two heavily pretreated patients with recurrent breast cancer had minor responses to rhizoxin, one at 1.6 mg m$^{-2}$ and the other at 2.6 mg m$^{-2}$. Plasma concentrations of rhizoxin were measured by high-performance liquid chromatography and showed considerable inter-subject variation in the plasma concentration–time profiles; the area under the curve ranged from 0.29 to 0.96 $\mu$g ml$^{-1}$ min$^{-1}$. Rhizoxin has shown some clinical activity in the phase I study and a dose of 2.0 mg m$^{-2}$ administered by i.v. bolus every 3 weeks was recommended for phase II studies (Bissett *et al.*, 1992). The Cancer Research Campaign Phase II Trials Committee therefore initiated three studies to assess the efficacy of rhizoxin in the treatment of ovarian, renal and colorectal cancer, in collaboration with the Early Clinical Trial Group of the EORTC which performed phase II trials in breast, head and neck and non-small-cell lung cancer using the same protocol.

### Patients and methods

Eligibility criteria common to the three studies were as follows: histological diagnosis of ovarian, colorectal and renal cancer; performance status (WHO scale) $\leqslant 2$; life expectancy $>3$ months; white blood count (WBC) $>4000$ mm$^{-3}$; platelets $>100\,000$ mm$^{-3}$; creatinine $\leqslant 150$ $\mu$mol l$^{-1}$; bilirubin $<20$ $\mu$mol l$^{-1}$; uni- or bidimensionally measurable lesions with documented progression within 2 months before the study. Previous immunotherapy but not chemotherapy was permissable in patients with renal cancer. In ovarian cancer, patients should have received no more than one previous chemotherapy regimen in the 12 months before entry and no chemotherapy during the 4 months before entry. Colorectal cancer patients may not have received more than one chemotherapy regimen for advanced disease.

The drug was provided by Fujisawa Pharmaceutical (Japan) and was provided as a vacuum dried powder (5 mg of rhizoxin, 25 mg of mannitol and 25 mg of ascorbic acid) in a duopack which contains special diluent vials [2.5 ml of diluent, 80% (v/v) propylene glycol and 20% (v/v) ethanol]. The vial of rhizoxin was dissolved in 2.5 ml of special diluent and then 2.5 ml of sterile water was added giving a stock solution of 1 mg ml$^{-1}$, stable for 8 h at room temperature. Rhizoxin was administered by i.v. bolus into a peripheral vein at 2 mg m$^{-2}$ every 3 weeks. When treatment had to be delayed for 1 week because of myelosuppression (WBC $<3.0$, platelets $<100$), the next course was given at 75% previous dose. Similarly, for patients who developed $>$ grade 3 toxicity other than haematological, the decision to have therapy withheld or reduced to 75% was left to the investigators' discretion. Patients were evaluable for response after two courses of therapy; treatment was discontinued in instances of progressive disease while its continuation was left to the discretion of the investigator when there was no significant change in tumour size. Objective UICC criteria were used to assess response for measurable disease; non-measurable disease was not used to assess response. CA125 was used as a subsidiary measure of response. Patients in response remained on study until disease progression or excessive toxicity. The CTC criteria for toxicity and UICC criteria for response (in terms of target lesion size etc.) were applied (Miller *et al.*, 1981). All patients gave informed consent in this multicentre study and the trial protocols received local ethics committee support.

### Results

### *Ovarian cancer*

Twenty-two patients with progressive epithelial ovarian cancer resistant to conventional chemotherapy were entered in the study (Table I).

Five patients (two indications in one patient) were regarded as ineligible for the following reasons: indicator lesion previously irradiated (three patients), chemotherapy within previous 4 weeks (one patient), endocrine therapy within previous 4 weeks (one patient), previous malignancy at another site (one patient). Of the 17 eligible women the median age was 59 (range = 44–72 years), nine had a performance status (PS) of 0, six a PS of 1 and two a PS of

Correspondence: DJ Kerr
Received 19 December 1994; revised 13 April 1995; accepted 21 April 1995

Table I Characteristics of eligible patients

| | Ovarian cancer (n = 17) | Colorectal cancer (n = 18) | Renal cancer (n = 18) |
|---|---|---|---|
| Characteristics | | | |
| Median age (years) | 59 | 63 | 55 |
| Age range | 44–72 | 36–70 | 35–70 |
| Men/women | 0/17 | 12/6 | 12/6 |
| Performance status[a] | | | |
| 0 | 9 | 6 | 4 |
| 1 | 6 | 8 | 10 |
| 2 | 2 | 3 | 4 |
| Sites of disease | | | |
| Primary tumour | 11 | 4 | 11 |
| Local recurrence | 4 | 7 | 2 |
| Metastatic nodes | 6 | 3 | 6 |
| Lung | 2 | 4 | 13 |
| Liver | 7 | 12 | 2 |
| Previous treatment | | | |
| Surgery | 16 | 18 | 12 |
| Radiotherapy | 1 | 2 | 4 |
| Immunotherapy | 1 | 3 | 3 |
| Chemotherapy | 17 | 13 | 0 |
| None | 0 | 0 | 4 |

[a]Performance status not known in one patient.

2. Twelve patients had had one prior platinum-based chemotherapy regimen. Four had received two and one patient three different regimens (one of which contained taxol). In addition one patient had received interferon and two, hormonal therapy. Fifty-six cycles of rhizoxin were delivered to the 17 eligible patients (range = 1–7 cycles). The dose was not modified for any of the courses and treatment delays were minimal (four patients, 1–3 days) and not for reasons of toxicity.

Rhizoxin was fairly well tolerated and the most common toxicities included alopecia in 96% of courses (grade 1 and 2); lymphocytopenia in 78% of courses (grade 3, 25%); fatigue in 48% of courses (grade 3, 4%); stomatitis in 29% of courses (grade 3, 0%).

One patient who had received platinum-based chemotherapy previously had a partial response in a pelvic mass and para-aortic lymphadenopathy which was apparent after two cycles of chemotherapy with a duration of 3 months. No patients had a separate response using the CA125 criteria of the study.

### *Colorectal cancer*

Twenty patients with advanced colorectal cancer were entered in the study (Table I). Two patients were ineligible because they did not fulfil the entry criteria and are not included for analysis. Seventeen of the 18 eligible patients received at least two courses of rhizoxin 2 mg m$^{-2}$ at 3 weekly intervals. Fourteen of the 18 eligible patients were WHO performance status 0 or 1. Four patients had more than three sites of disease. A total of 58 courses were given, with the mean number being three.

The most common toxicities were alopecia in 91% of courses (grades 1 and 2); tiredness in 45% of courses (grade 3, 5%); lymphocytopenia in 38% of courses (grade 3, 9%) and stomatitis in 21% of courses (grade 3, 3%). Pain in tumour-associated sites after administration of rhizoxin was noted in three patients. Complete response in two liver lesions was reported in one patient but disease progression at other sites. Overall, therefore, no responses were seen.

### *Renal cancer*

Twenty patients with advanced renal cancer were entered in the study (Table I). Two patients were ineligible because they did not fulfil the entry criteria and are not included for analysis. The 18 eligible patients all received at least two courses of rhizoxin 2 mg m$^{-2}$ at 3 weekly intervals. Fourteen of the 18 eligible patients were WHO performance status 0 or 1. Eleven of the patients still had a primary renal carcinoma *in situ* and six patients had more than three sites of disease. A total of 56 courses were given with the mean number being three. One patient was entered with a serum creatinine $>150\,\mu$mol l$^{-1}$ but an EDTA clearance was 55 ml min$^{-1}$ and was therefore eligible. This patient was the only patient who required dosage reductions because of toxicity. Interestingly, the only other patient with a serum creatinine $>140\,\mu$mol l$^{-1}$ did not suffer any WBC toxicity. Only one course of treatment was delayed for clinical reasons (chest infection).

The most common side-effects were alopecia in 96% of courses (grade 1 and 2); tiredness in 77% of courses (grade 3, 4%); diarrhoea in 41% of courses (grade 3, 4%); nausea and vomiting in 30% of courses (grade 3, 4%); fall in lymphocyte count in 80% of courses (grade 3 and 4, 27%); and stomatitis in 30% of courses (grade 3, 0%). No tumour responses were seen.

### Discussion

The results of these studies with only one partial response documented in ovarian cancer, suggests that at the tested schedule of 2 mg m$^{-2}$ administered by i.v. bolus every 3 weeks, rhizoxin has no clinically relevant anti-tumour activity in ovarian, colorectal or renal cancer. Similar patterns of toxicity were seen in each of the three groups of patients with moderate alopecia, lymphocytopenia and gastrointestinal toxicity as previously described in the phase I study (Bissett *et al.*, 1992).

**Acknowledgements**

The authors have performed this study under the auspices of the Cancer Research Campaign, Phase I and II Committees, and would like to thank Suzanne Witcomb for typing the manuscript.

### References

BISSETT D, GRAHAM MA, SETANOIANS A, CHADWICK GA, WILSON P, KOIER I, HENRAR I, SCHWARTSMANN G, CASSIDY J, KAYE SB AND KERR DJ. (1992). Phase I and pharmacokinetic study of rhizoxin. *Cancer Res.*, **52**, 2894–2899.

MILLER AB, HOOGSTRATEN B, STAQUET M AND BERARD D. (1981). Reporting results of cancer treatment. *Cancer*, **47**, 207–214.

TAKAHASHI M, IWASAKI S, KOBAYASHI H, OKUDA S, MURAI T AND SAITO Y. (1987). Rhizoxin binding to tubulin at the maytansine-binding site. *Biochim. Biophys. Acta*, **926**, 215–223.

TSURUO T, OH-HARA T, IIDA H, TSUKAGOSHI S, SATO Z, MATSUDI I, IWASAKI S, OKUDA S, SHIMIZU F, SASAGAWA K, FUKAMI M, FUKUDA K AND ARAKAWA M. (1986). Tumour cells and their vincristine-resistant sublines. *Cancer Res.*, **46**, 381–385.